# Clinical Significance of Serum MMP-9, S100-β and GFAP in Patients with Mental Disorders after Traumatic Brain Injury

**DOI:** 10.62641/aep.v53i1.1805

**Published:** 2025-01-05

**Authors:** Lijun Wu, Menghao Jin

**Affiliations:** ^1^Department of Laboratory, Wenzhou Hospital of Integrated Traditional Chinese and Western Medicine, 325000 Wenzhou, Zhejiang, China; ^2^Department of Neurosurgery, Wenzhou Hospital of Integrated Traditional Chinese and Western Medicine, 325000 Wenzhou, Zhejiang, China

**Keywords:** craniocerebral trauma, mental disorders, matrix metalloproteinase-9, S100-β protein, glial fibrillary acidic protein

## Abstract

**Background:**

Diagnosing psychiatric disorders following craniocerebral trauma primarily depends on clinical symptoms and neuropsychological evaluation, which can be subjective and limited. This study aimed to investigate the diagnostic value of serum matrix metalloproteinase-9 (MMP-9), S100 calcium-binding protein β (S100-β), and glial fibrillary acidic protein (GFAP) in post-traumatic mental disorders.

**Methods:**

A retrospective analysis was conducted on 108 patients with craniocerebral trauma admitted to Wenzhou Hospital of Integrated Traditional Chinese and Western Medicine between January 2021 and December 2023. Patients were categorized into a post-traumatic mental disorder group (n = 68) and a simple craniocerebral trauma group (n = 40) according to whether they had mental disorders. Serum MMP-9, S100-β, and GFAP levels were measured using enzyme-linked immunosorbent assay (ELISA) and compared between the two groups. Logistic multivariate regression identified risk factors for post-traumatic mental disorders, while receiver operating characteristic (ROC) curve analysis assessed the predictive value of the biomarkers. Spearman correlation analysis examined the relationship between serum biomarkers and the presence of post-traumatic mental disorders.

**Results:**

Serum levels of MMP-9, S100-β, and GFAP were significantly elevated in the post-traumatic mental disorder group compared to the simple traumatic brain injury group (*p* < 0.001). Logistic regression revealed that craniocerebral injury severity, family satisfaction, and serum levels of S100-β and GFAP were significant risk factors for post-traumatic mental disorders (*p* < 0.05). The areas under the ROC curve for MMP-9, S100-β, and GFAP were 0.768, 0.937, and 0.904, respectively. Spearman correlation analysis showed that serum MMP-9, S100-β and GFAP were significantly positively correlated with the incidence of post-traumatic mental disorders (*p* < 0.001).

**Conclusion:**

The levels of MMP-9, S100-β and GFAP were abnormal in the serum of patients with craniocerebral trauma. These biomarkers hold significant diagnostic value in patients with post-traumatic stress disorder.

## Introduction

Traumatic brain injury (TBI) is a prevalent and severe neurological condition that not only causes direct physical damage but also predisposes patients to a range of mental disorders, including anxiety, depression, and cognitive dysfunction. These complications significantly impair the quality of life of the patient and hinder social function recovery [1]. Therefore, identifying effective diagnostic markers is critical for early detection, accurate diagnosis, and timely treatment of post-traumatic mental disorders [2].

Following TBI, significant alterations occur in brain structure and function, with damaged brain tissue releasing various biomolecules that may contribute to the occurrence and development of mental disorders [3]. Research indicates that individuals with TBI are at a significantly higher risk of developing psychiatric disorders than the general population, with the severity of mental disorders correlating with the severity of brain injury [4]. However, the pathogenesis of post-traumatic mental disorders remains poorly understood, posing challenges for diagnosis and treatment [5].

In recent years, serum biomarkers have gained attention in diagnosing neurological diseases. Among these, matrix metalloproteinase-9 (MMP-9), S100 calcium-binding protein β (S100-β), and glial fibrillary acidic protein (GFAP) have emerged as key markers due to their specific expression in brain injury [6, 7, 8].

MMP-9, a protease involved in degrading extracellular matrix, is significantly elevated after TBI and may contribute to blood-brain barrier disruption and inflammatory responses. Elevated MMP-9 levels degrade extracellular matrix components, compromising the blood-brain barrier and allowing harmful substances in the blood to enter the brain parenchyma, exacerbating neural damage. Additionally, MMP-9 promotes inflammatory cell infiltration, releasing inflammatory mediators that impair neuronal function, increasing the risk of post-traumatic mental disorders [9].

S100-β, a calcium-binding protein primarily secreted by astrocytes, is released into the blood stream following brain damage. Its serum levels correlate with the degree of brain injury, and elevated concentrations of S100-β are neurotoxic, inducing neuronal apoptosis and impairing neuron survival and function. S100-β also activates microglia, triggering an inflammatory response that exacerbates neural damage, potentially leading to mental disorders such as anxiety, depression, and cognitive dysfunction [10].

GFAP is an astrocyte-specific marker. Following TBI, GFAP is released from damaged glial cells, and its serum levels reflect the severity of brain injury. Elevated GFAP indicates astrocyte activation, which can release inflammatory factors and neurotoxic substances, further damaging neurons. GFAP also plays a role in glial scar formation, which may disrupt neural circuit reconstruction and hinder functional recovery, contributing to mental disorders. In addition, GFAP may interact with other neurotransmitter systems, disrupting neural signal transmission and worsening psychiatric symptoms [11].

Although these serum markers have been studied in traumatic brain injury, their precise diagnostic value for post-traumatic mental disorders remains unclear. This study aimed to explore the diagnostic potential of serum MMP-9, S100-β, and GFAP in post-traumatic mental disorders and provide a foundation for clinical diagnosis and treatment. By measuring the levels of these markers in the serum of patients, combined with clinical and neuropsychological assessments, our findings establish a reliable and convenient diagnostic method for the early identification of TBI patients at risk of mental disorders, enabling timely and targeted interventions to improve patient outcomes.

## Materials and Methods

### General Information

This study retrospectively analyzed patients with craniocerebral trauma admitted to Wenzhou Hospital of Integrated Traditional Chinese and Western Medicine between January 2021 and December 2023. Eligible patients were included based on the following criteria: ① a confirmed history of craniocerebral trauma before admission; ② clinical diagnosis of craniocerebral injury upon admission; ③ ability to communicate and comprehend; ④ no history of mental illness; ⑤ no congenital intellectual hypoplasia; ⑥ no neurological disorders; ⑦ complete medical records and laboratory data [12]. Exclusion criteria included a history of mental illness, primary intellectual disability, or central nervous system diseases. A total of 108 patients meeting these criteria were divided into post-traumatic mental disorder group (n = 68) and simple traumatic brain injury (TBI) group (n = 40) according to whether the patients had mental disorders.

In research on post-traumatic brain disorder involving biomarkers such as serum MMP-9, S100-β, and GFAP, the Declaration of Helsinki is strictly followed, ethical approval is obtained, and patients are ensured to participate in the research with full informed consent, so as to guarantee the legitimacy, scientific and ethical nature of the research.

### Detection Methods of Serum MMP-9, S100-β, and GFAP

Serum MMP-9 (DMP900, R&D Systems, Minneapolis, MN, USA), S100-β (ASB-OKCD06506, Enzo Life Sciences, Farmingdale, NY, USA) and GFAP (MBS733397, Cambridge, MA, USA) were detected by enzyme-linked immunosorbent assay (ELISA, MyBioSource Inc., San Diego, CA, USA). And was measured using an enzyme label (Thermo Fisher Scientific, Waltham, MA, USA).

The ELISA procedure was as follows: distilled water, sample testers, an oscillator, and a magnetic stirrer were prepared. The required reagents were mixed, and standard and blank wells were established. In each reaction well, 50 µL of the specialized diluent and 50 µL of the test sample were added, followed by 50 µL of biotin-labeled antibody. The plates were sealed and incubated at 37 °C for 1 hour. After incubation, the wells were washed with washing buffer three times, and excess liquid was blotted with absorbent paper. Subsequently, 80 µL of the prepared affinity streptomycin-HRP was added to each reaction well, gently mixed, and incubated at 37 °C for 30 minutes. After further washing, 50 µL each of substrate A and substrate B were added to the wells, and the plates were incubated at 37 °C for 10 minutes. The reaction was terminated by adding 50 µL of stop solution, and the optical density (OD) was determined at 450 nm. Serum levels of MMP-9, S100-β, and GFAP were calculated using the standard curve generated from known concentrations of standards.

### Statistical Methods

Data were analyzed using SPSS Version 26.0 (Version: 26.0, manufacturer: International Business Machines Corporation, Headquarters: Armonk, NY, USA). Measurement data were measured for normality and homogeneity of variance and were expressed as mean ± standard deviation ($\bar{x}$ ± *s*). Categorical data were presented as percentages (%) and compared using the Chi-square test. Differences between measurement data were analyzed using the *t*-test. Spearman correlation analysis was conducted to assess the relationship between serum MMP-9, S100-β, and GFAP levels and the incidence of post-traumatic mental disorders. Multivariate logistic regression was used to identify risk factors for post-traumatic mental disorders, and the diagnostic value of serum biomarkers was evaluated using receiver operating characteristic (ROC) curve analysis. A *p*-value < 0.05 was considered statistically significant.

## Results

### Comparison of General Clinical Data between the Simple Traumatic Brain Injury Group and the Post-Traumatic Mental Disorder Group

Significant differences were observed between the simple traumatic brain injury (TBI) group and the post-traumatic mental disorder group in terms of family satisfaction and severity of brain injury (*p* < 0.001). There was no significant difference in age, gender, and combined with other obstacles between the two groups (all *p* > 0.05), as shown in Table 1.

**Table 1.** **Comparison of general clinical data between the two groups**.

Index		Simple craniocerebral trauma group (n = 40)	Post-traumatic mental disorder group (n = 68)	χ ^2^ */t*	*p*-value
Age (years)		40.53 ± 5.13	40.63 ± 5.18	0.104	0.917
Gender (n)				0.018	0.893
	Male	23	40		
	Female	17	28		
Family satisfaction (n)				13.202	<0.001
	Satisfied	33	32		
	Dissatisfied	7	36		
Craniocerebral injury (n)				36.462	<0.001
	Mild	29	10		
	Moderate-severe	11	58		
Combined with other obstacles (n)				0.408	0.523
	Yes	11	15		
	No	29	53		

Note: χ^2^ is the Chi-square test; *t* is the independent sample *t*-test. Combined with other disorders include motor disorders and sensory disorders.

### Comparison of Serum MMP-9, S100-β, and GFAP Levels between the Simple TBI Group and the Post-Traumatic Mental Disorder Group

**Fig. 1.**
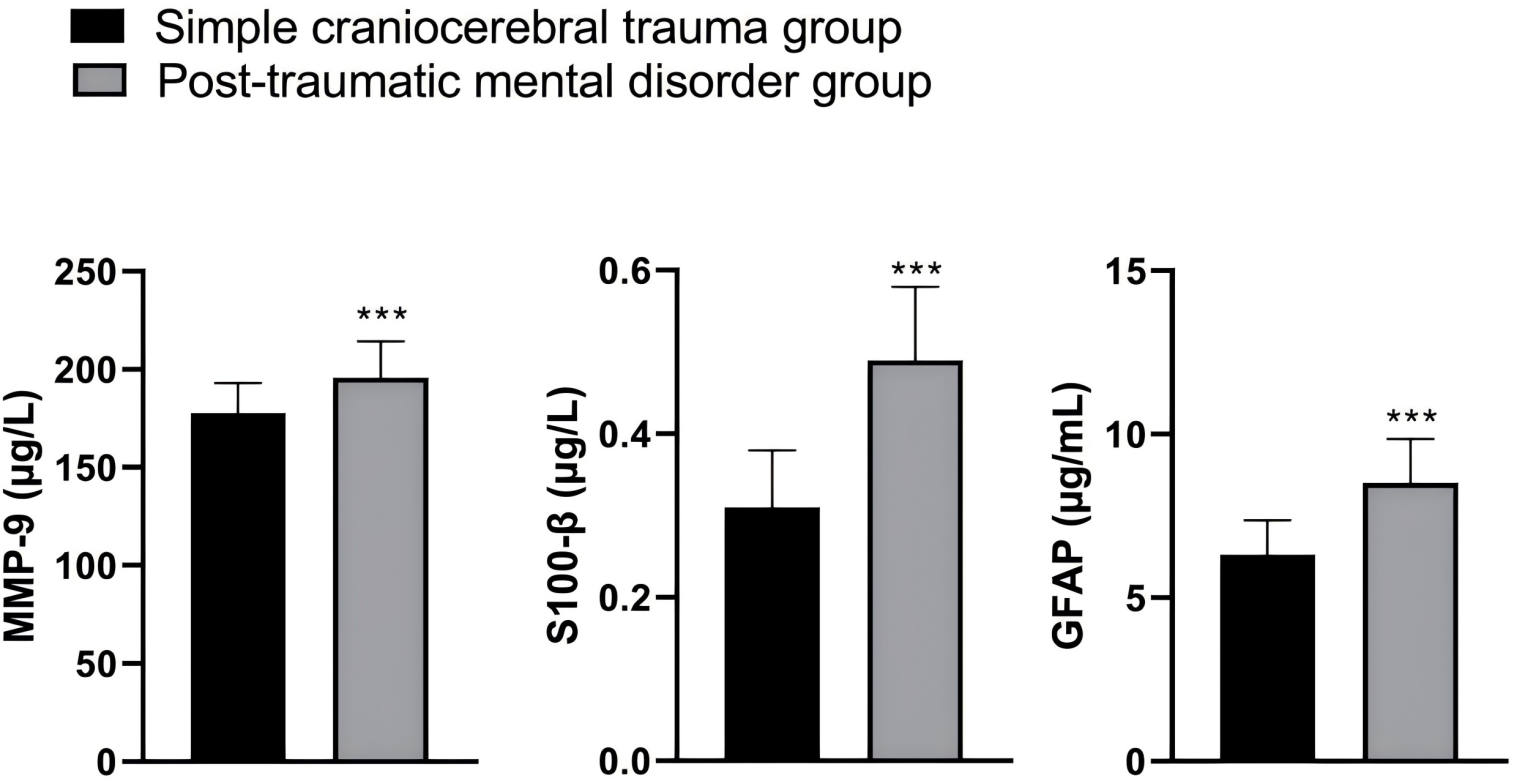
**Comparison of serum levels of MMP-9, S100-β, and GFAP between the two groups**. ***p < 0.001 compared to the simple craniocerebral trauma group. Note: MMP-9, matrix metalloproteinase-9; S100-β, S100 calcium-binding protein β; GFAP, glial fibrillary acidic protein.

The serum levels of MMP-9, S100-β, and GFAP were significantly higher in the post-traumatic mental disorder group compared to the simple TBI group, with all differences reaching statistical significance (*p* < 0.001). These findings are detailed in Table 2 and Fig. 1.

**Table 2.** **Comparison of serum MMP-9, S100-β, and GFAP levels between the two groups**.

Group	MMP-9 (µg/L)	S100-β (µg/L)	GFAP (µg/mL)
Simple craniocerebral trauma group (n = 40)	177.63 ± 15.46	0.31 ± 0.07	6.31 ± 1.06
Post-traumatic mental disorder group (n = 68)	195.83 ± 18.52	0.49 ± 0.09	8.51 ± 1.35
*t*	5.234	10.699	8.812
*p*-value	<0.001	<0.001	<0.001

Note: *t* is the independent sample *t*-test. MMP-9, matrix metalloproteinase-9; S100-β, S100 calcium-binding protein β; GFAP, glial fibrillary acidic protein.

### Logistic Multivariate Analysis of Risk Factors for Post-Traumatic Mental Disorders

**Fig. 2.**
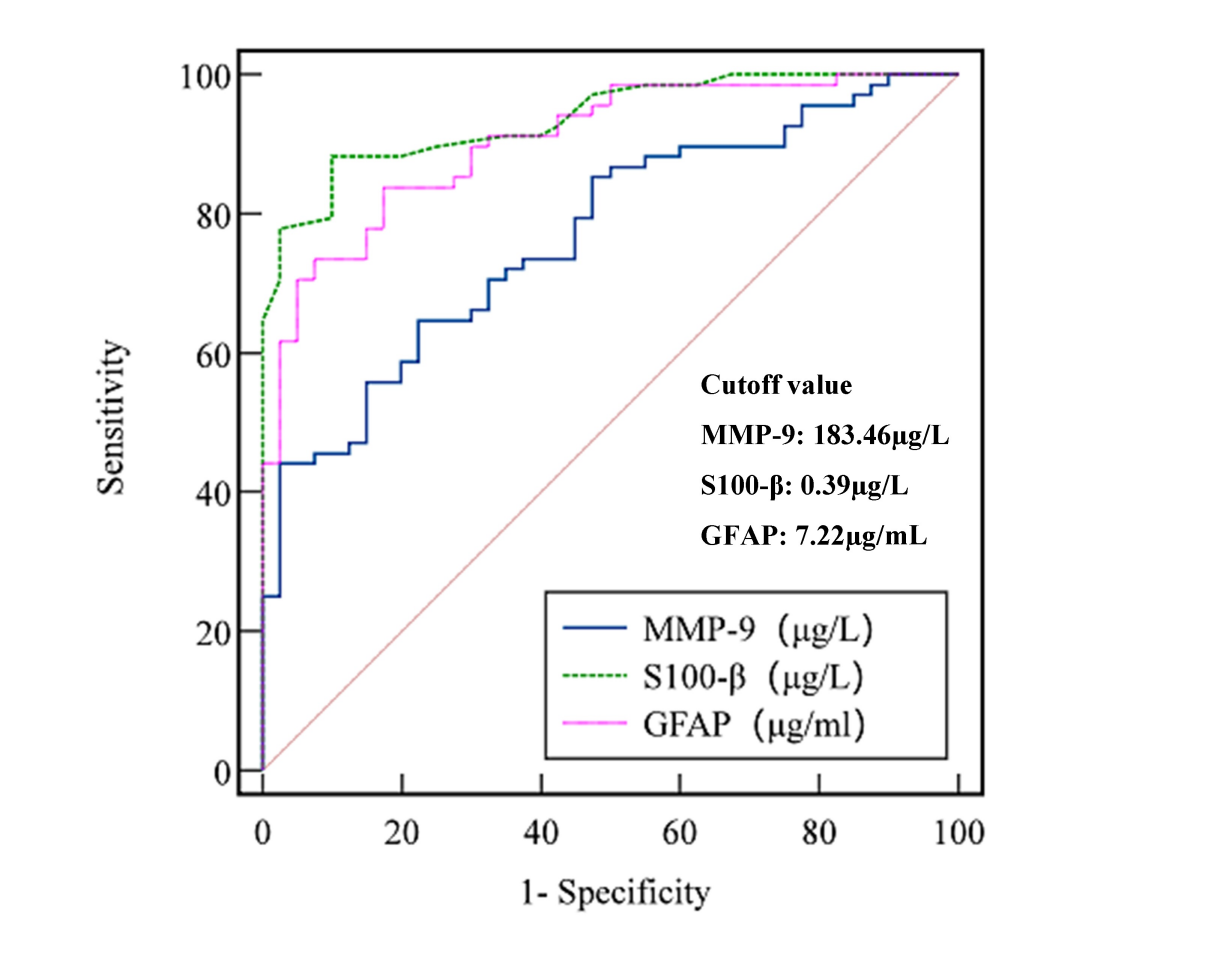
**Receiver operating characteristic (ROC) curve analysis of serum MMP-9, S100-β, and GFAP for the diagnosis of post-traumatic mental disorders**.

Multivariate logistic regression analysis was conducted to identify risk factors for post-traumatic mental disorders (Table 3). The occurrence of post-traumatic mental disorder was taken as the dependent variable, while family satisfaction, the severity of traumatic brain injury, and serum levels of MMP-9, S100-β, and GFAP were included as independent variables. Serum MMP-9, S100-β, and GFAP levels were categorized based on their mean values, which were 189.08 µg/L, 0.42 µg/L, and 7.69 µg/mL, respectively. The results of the logistic regression model showed that severity of craniocerebral injury, family satisfaction, and serum levels of S100-β and GFAP were significant risk factors for post-traumatic mental disorders (*p* < 0.05), as shown in Table 4 and Fig. 2.

**Table 3.** **Assignment scale for logistic multivariate analysis**.

Factor	Assignment
Dependent variable	Y
Mental disorder after craniocerebral trauma	Did not occur = 0; Occurred = 1
Independent variable	X
Family satisfaction	Satisfied = 0; Dissatisfied = 1
Degree of craniocerebral injury	Mild = 0; Moderate to severe = 1
MMP-9	≤189.08 µg/L = 0; >189.08 µg/L = 1
S100-β	≤0.42 µg/L = 0; >0.42 µg/L = 1
GFAP	≤7.69 µg/mL = 0; >7.69 µg/mL = 1

**Table 4.** **Logistic multivariate analysis of risk factors for post-traumatic mental disorders**.

Index	β	SE	Wald	OR	95% CI	*p*-value
MMP-9	1.834	1.063	2.976	6.261	0.779∼50.322	0.085
S100-β	4.606	1.312	12.331	100.044	7.653∼1307.888	0.000
GFAP	4.907	1.441	11.599	135.206	8.029∼2276.874	0.001
Family satisfaction	3.084	1.385	4.956	21.840	1.446∼329.907	0.026
Degree of craniocerebral injury	2.599	0.996	6.814	13.455	1.911∼94.726	0.009

SE, standard error; OR, odds ratio; CI, confidence interval.

### Diagnostic Value of Serum MMP-9, S100-β, and GFAP in Post-Traumatic Mental Disorders

The areas under the receiver operating characteristic (ROC) curves (AUC) for serum MMP-9, S100-β, and GFAP in the diagnosis and evaluation of mental disorders in patients with craniocranial trauma were all greater than 0.70, showing high specificity and sensitivity. The AUC values for MMP-9, S100-β, and GFAP were 0.768, 0.937, and 0.904, respectively (*p* < 0.05), as shown in Table 5 and Fig. 2.

**Table 5.** **Diagnostic value of serum MMP-9, S100-β, and GFAP in post-traumatic mental disorders**.

Index	Sensitivity (%)	Specificity (%)	AUC	95% CI	Youden index	Cut-off value
MMP-9	64.71	77.50	0.768	0.677∼0.844	0.422	183.46 µg/L
S100-β	88.24	90.00	0.937	0.873∼0.975	0.694	0.39 µg/L
GFAP	83.82	82.50	0.904	0.832∼0.952	0.646	7.22 µg/mL

AUC, areas under the receiver operating characteristic curves; CI, confidence interval.

### Relationship between Serum MMP-9, S100-β, and GFAP Levels and the Onset of Post-Traumatic Mental Disorders

Spearman correlation analysis revealed that serum MMP-9, S100-β, and GFAP levels were significantly positively correlated with the incidence of post-traumatic mental disorders (all *p* < 0.001), as shown in Table 6.

**Table 6.** **Spearman correlation analysis of serum MMP-9, S100-β, GFAP, and the incidence of post-traumatic mental disorders**.

Index	Mental disorder after craniocerebral trauma	
	*r*	*p*-value
MMP-9	0.449	<0.001
S100-β	0.731	<0.001
GFAP	0.675	<0.001

Note: *r* is the correlation parameter test.

## Discussion

Craniocerebral trauma is a common injury that not only causes direct physical harm but also poses a risk of developing mental disorders, which can severely impact the quality of life and social functioning of patients. Understanding the pathogenesis and the need for timely diagnosis is crucial for improving patient prognosis. The pathogenesis of post-traumatic mental disorders is multifaceted. Trauma can directly damage key brain regions, such as the frontal and temporal lobes, closely related to emotion, cognition, and behavior regulation. Damage to these regions disrupts neurotransmitter transmission and metabolism, leading to imbalances in dopamine, 5-hydroxyserotonin, and other key neurotransmitters, which contribute to the development of psychiatric symptoms [13]. Moreover, psychological and social factors must not be overlooked. Traumatic brain injury often brings a double blow to patients, both physically and psychologically, resulting in emotional responses such as anxiety and depression. Without sufficient social support and psychological intervention, these negative emotions can persist and worsen, ultimately manifesting as mental disorders [14, 15].

Matrix metalloproteinase-9 (MMP-9) is an enzyme that degrades extracellular matrix components. Following traumatic brain injury, damaged brain tissue triggers an inflammatory cascade, leading to a significant upregulation of MMP-9. Overexpression of MMP-9 compromises the integrity of the blood-brain barrier, allowing substances typically isolated from the central nervous system to infiltrate the brain, triggering a series of immune reactions and neural damage [16, 17]. In this study, the post-traumatic mental disorder group exhibited higher serum MMP-9 levels compared to the simple brain trauma group, identifying MMP-9 as a risk factor. The destruction of the blood-brain barrier alters the microenvironment of the brain, affecting neuronal function and survival. Harmful substances such as inflammatory cytokines and free radicals infiltrate brain tissue, further exacerbating neuronal injury and death [18]. This neuronal damage, especially in areas related to emotion, cognition, and behavior, may lead to symptoms such as anxiety, depression, and cognitive impairment in patients [19]. Furthermore, MMP-9 may promote the activation and proliferation of glial cells, which release inflammatory mediators and neurotoxins, amplifying neuroinflammatory responses. This chronic neuroinflammation can result in secondary neuronal damage, long-term accumulation of neuroinflammation and neuronal damage, and increasing the risk of developing post-traumatic mental disorders [20].

S100-β is a calcium-binding protein primarily secreted by glial cells and plays a significant role in the pathophysiological processes following traumatic brain injury. Brain trauma damages glial cells, releasing large amounts of S100-β into the peripheral blood [21, 22]. This study found that serum S100-β level was abnormally elevated in patients with post-traumatic mental disorders, identifying it as an independent risk factor. The elevated levels of S100-β likely reflect the severity of brain injury, and its presence in high concentrations may contribute to the onset of mental disorders through various mechanisms. On the one hand, excessive S100-β has neurotoxic effects; it induces oxidative stress, producing free radicals that damage neuronal membranes, organelles, and DNA, leading to neuronal dysfunction or death. The loss of neurons disrupts neural circuits and information transmission, negatively affecting emotional regulation and cognitive function, which may manifest as psychiatric symptoms. On the other hand, S100-βactivates inflammatory pathways, stimulating microglia and astrocytes to release pro-inflammatory cytokines, such as tumor necrosis factor-α (TNF-α) and interleukin-1β (IL-1β), further exacerbating neuroinflammation and contributing to the onset of mental disorders [21, 23]_._

GFAP is a signature protein of astrocytes, and its serum levels are closely related to the development of post-traumatic mental disorders. Following traumatic brain injury (TBI), astrocytes undergo mechanical damage and stimulation, releasing large quantities of GFAP into the bloodstream [24]. The elevated serum GFAP reflects the degree of astrocytic injury and its activation status. Astrocytes play a key role in maintaining brain homeostasis, supporting neurotransmitter metabolism, and providing nutritional support to neurons. Damage to astrocytes disrupts these essential functions, compromising brain stability.

In this study, patients with post-traumatic mental disorders exhibited significantly higher GFAP levels compared to those with simple craniocerebral trauma. This increase is likely due to impaired neurotransmitter uptake and metabolism by astrocytes in these patients, leading to imbalances in key neurotransmitters, such as dopamine and 5-hydroxytryptamine, which are strongly associated with psychiatric symptoms like anxiety and depression [25]. Additionally, injured astrocytes may release harmful substances, including excitatory amino acids, exacerbating neuronal damage. Such neuronal injury and dysfunction disrupt neural connectivity and information transmission, impairing cognitive, emotional, and behavioral functions.

Studies have shown that persistently elevated serum GFAP levels after craniocerebral trauma injury are associated with a higher risk of psychiatric symptoms, including cognitive impairment and emotional instability. Moreover, changes in GFAP levels can serve as a pivotal biomarker for evaluating treatment efficacy and predicting patient prognosis [26].

In addition to serum MMP-9, S100-β, and GFAP, other biomarkers like neuron-specific enolase (NSE) are also elevated after traumatic brain injury, reflecting the extent of neuronal damage. Each biomarker has unique characteristics. While NSE is highly specific for neuronal damage, it may also be elevated in other neurological disorders. MMP-9 primarily reflects blood-brain barrier disruption and inflammation, S100-β reflects glial cell injury and function, and GFAP specifically targets astrocyte activation. The combined use of these biomarkers provides a more comprehensive picture of the pathophysiological changes following craniocerebral trauma. The sensitivity and specificity of these biomarkers vary, but their combined application improves the accuracy and reliability of diagnosing post-traumatic mental disorders.

Serum levels of MMP-9, S100-β, and GFAP hold significant promise for the future diagnosis and management of post-traumatic mental disorders. Firstly, these biomarkers could serve as valuable tools for early diagnosis. Monitoring changes in serum levels of these indicators may help identify at-risk patients before psychiatric symptoms emerge, allowing for timely intervention. Secondly, these biomarkers can help assess the severity of TBI and mental disorders, aiding in the formulation of tailored treatment plans. Additionally, they can be used to monitor treatment efficacy; dynamic tracking of these biomarkers throughout therapy can provide insights into the success of interventions. Finally, as technology advances, the development of more rapid and precise detection methods is expected, improving diagnostic accuracy and enhancing patient care. These biomarkers will likely contribute to better diagnostic experiences and improved prognoses for patients with post-traumatic mental disorders.

## Conclusion

In patients with craniocerebral trauma, serum levels of MMP-9, S100-β, and GFAP were found to be significantly altered. The severity of craniocerebral injury, family satisfaction, and levels of serum S100-β and GFAP were identified as key risk factors for the development of post-traumatic mental disorders. The diagnostic value of MMP-9, S100-β, and GFAP in detecting post-traumatic mental disorders is substantial, with a significant correlation observed among the biomarkers. Given their diagnostic significance, these biomarkers hold potential for clinical use in identifying and managing post-traumatic mental disorders.

## Availability of Data and Materials

The data used to support the findings of this study are available from the corresponding author upon request.
